# Ceramic Dental Restorations—From Materials Sciences to Applications

**DOI:** 10.3390/ma18133116

**Published:** 2025-07-01

**Authors:** Han Chao Chang, Satoshi Yamaguchi

**Affiliations:** 1 National Center for Instrumentation Research, National Institutes of Applied Research, Hsinchu 30076, Taiwan; 2 Graduate School of Dentistry, The University of Osaka, Osaka 565-0871, Japan

In response to the growing demand from patients for enhanced oral aesthetics, as well as improved chewing and occlusion, coupled with advancements in CAD/CAM technology, a variety of dental ceramic materials have been developed over the past two decades to serve as alternatives to traditional alloys and pure metals. These materials include glass-ceramics, all-ceramic zirconia (ZrO_2_), and polymer-infiltrated ceramic networks, among others. Consequently, the objective of this Special Issue is to gather the most recent research findings on dental ceramic materials to identify the limitations of these materials, evidenced by fundamental research and clinical follow-up, and to explore viable solutions for addressing these shortcomings. Following an extensive period of eight months dedicated to invitations and peer review, seven articles authored by esteemed scholars from Saudi Arabia, South Korea, Germany, Egypt, Brazil, the United States, Switzerland, and Japan have been successfully published.

A multinational research team, under the leadership of Dr. Benalcázar-Jalkh [1] from the University of São Paulo, Brazil, conducted a retrospective study examining the use of polycrystalline ceramic materials in large-span fixed dental prostheses (FDPs). The team introduced a significant revision to the classification system for polycrystalline ceramics, superseding the previously established organizational framework for dental ceramic classification. They noted that glass-infiltrated ceramics produced through slip-casting, as well as pure alumina ceramics, have been withdrawn from the market, resulting in their removal from the commonly utilized materials list in dentistry. It is posited that augmenting the yttrium content will improve the stability of the cubic phase, thereby enhancing the translucency of zirconia crowns. However, this increase in the cubic phase may also influence the hydrothermal stability of zirconia as well as its mechanical properties. Research indicates that 3Y-TZP exhibits significant sensitivity to hydrothermal degradation and low-temperature degradation. In contrast, materials with a yttrium content of 4% or greater (≥4Y-PSZ) may not experience phase transformation following aging, which can be attributed to their elevated yttrium content and the predominance of cubic phases. The Benalcázar-Jalkh team asserts that each type of zirconia restorative material possesses a specific clinical application range. Translucent 3Y-TZP partial veneers may serve as a substitute for conventional 3Y-TZP veneers, thereby minimizing the likelihood of porcelain fracture. Additionally, 5Y-PSZ and multilayer zirconia are suitable for use in single crowns and large-span fixed dental prostheses (FDPs) on titanium artificial root abutments, respectively. Furthermore, integral multilayer zirconia (3Y-TZP/5Y-PSZ) can be dyed to enhance aesthetic outcomes and is applicable for full arch support restorations.

In in vitro studies, Dr. Kim from Yonsei University College of Dentistry [2] analyzed the effects of surface treatment with commercially available etching agents on the bond strength between zirconia and resin cement and compared them with those achieved using air abrasion alone. In the groups that did not undergo thermocycling, specimens that were surface-treated with a solution did not exhibit a statistically significant enhancement in shear bond strength compared to sandblasted samples. Conversely, in the groups subjected to thermocycling, smart-etched specimens demonstrated the highest shear bond strength. In the short term, the various etching agents did not yield a significant improvement in bond strength relative to sandblasting alone. The X-ray diffraction (XRD) analysis revealed the presence of a monoclinic phase structure in all three samples not subjected to thermocycling. Conversely, the monoclinic phase was absent in the zirconia sample that underwent a two-hour etching process with a strong acid. These findings indicate that pneumatic grinding or heat treatment can induce phase transformations in zirconia, whereas strong acid etching does not affect the phase composition of the zirconia surface.

Professor Zenthöfer and his team from the University of Heidelberg [3] conducted a study to assess the fracture resistance of 3D-printed zirconia occlusal veneers with varying thicknesses. They also examined the impact of different abutment materials on this resistance. Their research focused on both the total fracture resistance and the lowest contact force. Their study indicates that when the abutment comprised Co-Cr alloy, the Korean 3D printing method demonstrated superior total fracture resistance and the lowest contact force, particularly when compared to other 3D printing techniques and traditional milling processes. This advantage was observed across various wall thicknesses of 0.4 mm, 0.6 mm, and 0.8 mm. It is important to note that thinner wall thickness can compromise the fracture resistance of posterior tooth occlusal veneers. Therefore, it is advisable to avoid the clinical use of 3D-printed zirconia posterior tooth occlusal veneers with a wall thickness of 0.4 mm without appropriate limitations. An additional study conducted by the University of Heidelberg [4] examined the fracture resistance and fracture modes of tooth-supported conventional fixed dental prostheses (cFDPs) comprising various zirconia materials, specifically 3Y-TZP, 4Y-PSZ, and 5Y-PSZ, as well as differing framework thicknesses. The findings suggest that cFDPs constructed from 3Y-TZP exhibited fracture resistance surpassing the masticatory forces typically experienced in the posterior region. Conversely, the utilization of 4Y-PSZ or 5Y-PSZ for the fabrication of cFDPs in the posterior area is not advisable.

A collaborative research team, spearheaded by Mansoura University in Egypt [5], conducted a study to investigate the impact of ferrule design and pulpal extension on the fitting accuracy and fracture resistance of endocrowns made from zirconia-reinforced lithium silicate (ZLS) ceramic material. The endocrowns were prepared from ZLS blocks utilizing CAD/CAM milling technology. Following the cementation process, the specimens were subjected to thermal aging for 5000 cycles, and their marginal adaptation was subsequently assessed. Their study concluded that all designs, both with and without ferrule, as well as varying inlay depths, demonstrated clinically acceptable marginal and internal fit. Endocrowns lacking a ferrule exhibited a more conservative approach and greater fracture strength compared to designs featuring a 1-mm ferrule. Furthermore, an increase in inlay depth resulted in a notable enhancement in the fracture resistance of the 1-mm ferrule design.

The team of Dr. Attar [6] investigated how the repression of lithium disilicate glass-ceramics influences the shear bond strength of three distinct types of resin cements. The fundamental principle is that lithium disilicate ceramics, being glass-based and thus etchable, provide the benefits of adhesive cementation through the use of resin-based cements. The application of hydrofluoric acid to dental glass-ceramics results in the formation of tetrafluorosilane, which subsequently reacts with the glass-ceramic to generate soluble hydrofluorosilicic acid. Upon the removal of this acid, a uniform dental ceramic surface is achieved, characterized by an excellent porous micro-retentive texture. Research references have demonstrated that the adhesion of ceramic crowns utilizing resin cement significantly increases the fracture resistance of these crowns. They also identified numerous references indicating a substantial enhancement in flexural strength, attributed to the repeated pressing of lithium disilicate glass-ceramics. Taken together, their experiments demonstrated that the shear bond strength of samples utilizing Multilink N resin cement was notably superior to that of other samples across various cycles of pressing lithium disilicate glass-ceramics. Additionally, there was no cohesive failure of the resin cement observed in any of the three tests conducted. One possible explanation for the varying curing depths among the composite materials could be the relatively larger size of the filler particles (0.25–0.3 μm) in Multilink N, which may contribute to enhancing the strength of the resin cement.

In the realm of clinical research, Dr. Elkaffas [7] and his team carried out a two-year follow-up study through a randomized clinical trial, focusing on the efficacy of direct composite versus indirect ceramic laminate veneers in the treatment of multiple diastema closure cases. A group of 28 patients, with an average age of 26 years, underwent treatment involving 60 direct resin composite applications (Estelite Asteria; n = 14) and 60 indirect ceramic veneers (IPS e.max Press; n = 14) on their maxillary anterior teeth to address diastema closure. The veneers were assessed at the initial appointment and subsequently every six months for a duration of up to two years, utilizing the United States Public Health Service (USPHS) criteria for evaluation. In their study, researchers noted three instances of failure with indirect ceramic veneers, which included one case of debonding and two cases of fracture. Additionally, they found that four direct composite resin veneers (6.6%) exhibited moderate chipping and cracking after a period of 24 months. They also frequently observed staining (n = 11) and roughness (n = 14) in resin composite veneers. Ultimately, they found no significant difference in the survival rates between direct composite and ceramic veneers. Both materials effectively met aesthetic and functional standards; however, for anterior veneers, indirect ceramic laminate veneers were recommended as the preferred choice.

In the contributions to this Special Issue, encompassing narrative reviews, in vitro studies, and clinical trials, the zirconia family (3Y-TZP, 4Y-PSZ, and 5Y-PSZ) and lithium disilicate glass-ceramics are the primary dental ceramic materials under investigation. Several topics are addressed, including large-span FDPs, ceramic veneers, resin cements for bonding, ferrule design for endocrowns, and occlusal veneers. Furthermore, it can be inferred that a misalignment between clinical conditions and the selection of ceramic materials may result in damage or fracture of these materials. We are confident that these challenges will be addressed through the innovative efforts of dental materials scientists, ultimately enhancing patient chewing quality and extending the longevity of dental ceramic materials.
